# The Cost of Inadequate Sleep among On-Call Workers in Australia: A Workplace Perspective

**DOI:** 10.3390/ijerph15030398

**Published:** 2018-02-26

**Authors:** Grace E. Vincent, Irina Kinchin, Sally A. Ferguson, Sarah M. Jay

**Affiliations:** 1 School for Health, Medical and Applied Sciences, Central Queensland University, Adelaide 5034, Australia; i.kinchin@cqu.edu.au (I.K.); sally.ferguson@cqu.edu.au (S.A.F.); s.jay@cqu.edu.au (S.M.J.); 2 The Cairns Institute, James Cook University, Cairns 4870, Australia

**Keywords:** sleep, cost, workplace, impact, stand-by

## Abstract

On-call or stand-by is becoming an increasingly prevalent form of work scheduling. However, on-call arrangements are typically utilised when workloads are low, for example at night, which can result in inadequate sleep. It is a matter of concern that on-call work is associated with an increased risk of workplace injury. This study sought to determine the economic cost of injury due to inadequate sleep in Australian on-call workers. The prevalence of inadequate sleep among on-call workers was determined using an online survey, and economic costs were estimated using a previously validated costing methodology. Two-thirds of the sample (66%) reported obtaining inadequate sleep on weekdays (work days) and over 80% reported inadequate sleep while on-call. The resulting cost of injury is estimated at $2.25 billion per year ($1.71–2.73 billion). This equates to $1222 per person per incident involving a short-term absence from work; $2.53 million per incident classified as full incapacity, and $1.78 million for each fatality. To the best of our knowledge this is the first study to quantify the economic cost of workplace injury due to inadequate sleep in on-call workers. Well-rested employees are critical to safe and productive workplace operations. Therefore, it is in the interest of both employers and governments to prioritise and invest far more into the management of inadequate sleep in industries which utilise on-call work arrangements.

## 1. Introduction

On-call or stand-by is a form of scheduling whereby an employee can be called into work under emergency or unpredictable circumstances [1]. On-call arrangements typically allow for 24/7 work coverage, without the financial burden of providing 24/7 active coverage or when fixed rostering is not possible [1,2]. The industries that typically utilise on-call working arrangements include health care, emergency services, maintenance, and information technology [1]. In Australia and Europe, 20–25% of the workforce are required to do on-call work as part of their regular employment [3,4]. Furthermore, on-call work scheduling is becoming increasingly prevalent, for example from 2005 to 2015 the proportion of on-call workers in the United States (US) increased from 2% to 17% [3,5].

On-call arrangements are often utilised when workloads may be low; as such they typically occur outside of normal working hours, particularly at night [6]. When on-call periods occur overnight, there is likely to be a significant reduction in sleep [6]. For example, railroad engineers [7] and gas and electricity supply company supervisors [8] reported obtaining 0.5–1.5 h less sleep per night during on-call periods compared with not on-call. There is also evidence to suggest that being on-call, regardless of whether an employee is actually called, can also adversely impact sleep duration [9]. For example, in a study of hospital doctors, shorter sleep was reported when on-call compared with not on-call, even when no calls were actually received [10]. The major difference between on-call work and other irregular work schedules (e.g., shift work) is the inherent unpredictability of whether or not a worker will receive a call, and secondly whether or not they will be required to return to work. 

Adults should regularly sleep seven hours or more per night to support optimal health [11]. Inadequate sleep (less than seven hours) is associated with a plethora of adverse health impacts on a range of physiological and psychological functions, including neurobehavioural performance [12], metabolism [13], appetite regulation [14], and immune function [15]. It is particularly concerning that inadequate sleep is linked with an increased risk of workplace injuries. For example, workers with excessive daytime sleepiness (as a result of chronic inadequate sleep) or those who often reported trouble sleeping had a 1.40–2.20 fold increased risk of sustaining an occupational injury [16,17]. Furthermore, on-call nurses involved in weekly or more frequent ‘call ins’ to work have been found to have an almost two-fold increase in back and shoulder musculoskeletal injuries [18]. The relationship between working on-call, inadequate sleep, and injury risk is particularly concerning for on-call workers given the crucial roles (e.g., doctors, firefighters) these workers play in communities. 

Inadequate sleep impacts up to 40% of Australians at an annual economic cost of $66 billion, comprising $26.2 billion in financial costs (e.g., health system, productivity, informal care), and $40.1 billion in the loss of well-being [19]. Furthermore, the costs of other irregular work hours exert acute effects on sleep and alertness which can lead to considerable human and economic costs due to fatigue-related accidents and reduced productivity [20]. For example, road and workplace accidents, related to excessive sleepiness, are estimated to cost $71–93 billion per annum in the United States [21]. 

To the authors’ knowledge the economic cost of inadequate sleep in on-call work is yet to be investigated. On-call work is particularly common in industries where it is more cost-effective for an employer to have staff on-call at relevant times, rather than having employees rostered on when they are not needed. However, the economic benefits of implementing on-call arrangements should depend on worker health and safety risks being maintained at an acceptable level. As a first step into investigating the economic cost of on-call work, the objective of this study is to draw upon past research and validated costing methodology to quantify the economic cost of injury due to inadequate sleep of Australian on-call workers over one year.

## 2. Materials and Methods 

### 2.1. On-Call Survey

To assess the prevalence of inadequate sleep and determine the average age of on-call workers for the cost analysis, an online survey (Survey Monkey) was administered between June–August 2017. The survey was advertised to Australian on-call workers across a range of industries (e.g., healthcare, emergency services, hospitality, and information technology) via existing industry relationships, and social media. Interested participants were provided a link to the survey and were presented with an information sheet providing an outline to the study and stating that all responses were anonymous. Participants then completed a declaration confirming they were over eighteen years of age, residing in Australia, and had read and understood all information and provided consent to participate. Ethical approval was obtained from the Central Queensland University Ethics Committee (H17/05-88). Six participants did not explicitly indicate that they were over eighteen years old and/or resided in Australia. However, in consultation with our ethics committee, these participants were included in the final sample as they provided answers in the rest of the survey (e.g., how old are you?) that this information could be inferred from. 

The survey consisted of questions related to basic demographics (e.g., age, sex); on-call work patterns (e.g., work type, how often called); sleep; and levels and types of perceived stress as well as coping strategies. The majority of these data were not a focus of the current study and will be reported elsewhere. To determine the prevalence of inadequate sleep in Australian on-call workers, two questions were examined. The first was ‘how much sleep do you regularly get on weekdays (or work days), including naps?’. This question aimed to understand the prevalence of inadequate sleep in the on-call population. The second question was ‘how many hours of sleep do you regularly get when on-call, including naps?’. This allowed for a direct understanding of how on-call workers sleep, specifically during on-call periods. Responses were allocated into six sleep duration categories, <5 h, 5–6 h, 6–7 h, 7–8 h, 8–9 h, >9 h. Inadequate sleep was defined as a sleep duration below the recommended sleep duration of 7–9 h per night [11]; that is, the number of responses from the <5 h, 5–6 h, 6–7 h categories. 

### 2.2. Approach to Costing Estimation

**Figure 1:**
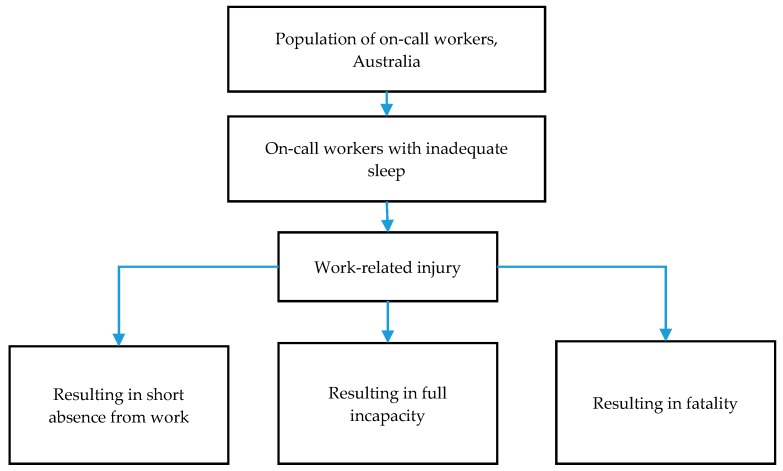
Flowchart of work-related injuries among on-call workers classified by severity.

The costing methodology utilized in this study was developed by the Industry Commission [22] to quantify the cost of work-related injury and illness for Australian employers, workers and the community. The same methodology was applied over the years by the Australian National Occupational Health and Safety Commission [23] and by Safe Work Australia [24,25]. This methodology was selected in the current study and deemed reliable and pertinent. The author (IK) also validated it in the past and quantified the cost of fatal and non-fatal suicide behavior [26,27]. Only workplace injuries were considered, regardless of shift type (i.e., on-call and not on-call). The framework presents both direct and indirect costs of work-related injuries segregated by severity. In line with the previous research [24,25,26,27], current analysis applies three levels of severity—“short absence from work”, “full incapacity”, and “fatality”. Short absence is described as “a minor work-related injury or illness, involving less than five working days absence from normal duties, where the worker was able to return to full duties. A work-related injury or disease, which results in the individual being permanently unable to return to work is classified as full incapacity. Fatality is a work-related injury or disease, which results in death” [24,25]. The cost of injury which did not result in any absence from work was not included in this study. Following Safe Work Australia [24,25], corresponding durations of absence (for use in calculation of production disturbance costs) are 0.2 weeks for short absence; and 2.6 weeks for full incapacity and fatality [24] (Figure 1). 

Consistent with the previous research [24,25,26,27], this costing analysis used six cost groups to derive the total cost of inadequate sleep among on-call workers: production disturbance costs, human capital costs, medical costs, administrative costs, other costs, and transfer costs (Table 1).

Our costing methodology adopts a workplace perspective. It builds on an incidence-based approach known as the lifetime cost approach. This approach provides an indicator of the benefits of reducing work-related incidents. The costs that an injury imposes in future years are discounted to present values (using 2016 CPI correction factor [28]). 

A further assumption made in the Safe Work Australia report [24], and carried over to this analysis, is that “the methodology is based on an ex-post approach in which costs are attributed to incidents after they occur and as a direct result of the incident. The total cost is estimated by aggregating the cost of each case and/or cost component from the bottom-up”. A summary of the key parameters, assumptions, and data sources for cost items is provided in Table 2.

### 2.3. Sensitivity Analysis Parameters

One-way deterministic sensitivity analyses were undertaken to test the robustness of results to changes in key parameters. We draw on the previous research [24,25,26,27] and consulted with experts in the field to derive upper and lower values for key parameters due to their uncertainty. The proportion of on-call workers with inadequate sleep on workdays (i.e., 66%) was reduced to 50%, as well as increased to 80% to reflect the fact that 80% of the included sample reported regularly obtaining inadequate sleep (i.e., <7 h) when on-call. The proportion of work-related injuries (i.e., 7.5%) was varied by 1.0 percentage points (±1.0%). The proportion of injuries resulting in full incapacity (i.e., 0.7%) was varied by 0.1 percentage points (±0.1%). The proportion of injuries resulting in fatality (i.e., 0.04 per 1000) was reduced to 0.02 per 1000. Following Safe Work Australia [24,25], mean age of injury (i.e., 42 years) was reduced to 40 years depicting the female mean age of injury. Average life expectancy at birth (i.e., 82.4 years) was reduced to 71.6 years and 77.0 years reflecting the average life expectancy of people born 40 and 42 years ago. Discount rate used to convert future costs to present value (i.e., 4.4%) was adjusted to 3% and 5%. Lastly, weighted average weekly earnings (i.e., $1317) were reduced to $1231 reflecting average weekly earnings of a gender-balanced population (50% male and 50% female).

## 3. Results

### 3.1. On-Call Survey: Inadequate Sleep

A total of 230 on-call workers provided consent to participate in the survey. Of these, 211 answered the three questions (average age, regular sleep duration on weekdays (workdays), and regular sleep duration while on-call) relevant for this analysis. The average age of on-call workers was 42 ± 12 years (mean ± SD). In this sample, 78% of workers had an on-call component as part of their main form of employment. The remaining 22% indicated that their on-call role was not part of their main form of employment (e.g., voluntary roles). 

Self-reported sleep duration in response to the questions, “how much sleep do you regularly get on weekdays (or work days), including naps?” and “how many hours of sleep do you regularly get when on-call, including naps?” are reported in Table 3. As such, 66% and 80% of the included sample reported regularly obtaining inadequate sleep (i.e., <7 h) on weekdays (or work days) and when on-call, respectively. 

### 3.2. Average Cost

Table 4 provides an overview of average cost associated with work-related injuries resulting in short absence, full incapacity, and fatality among on-call workers. The cost of an incident involving a short-term absence is estimated at $1222 per incident. The average cost of an incident resulting in full incapacity is $2.53 million per incident, and $1.78 million for each fatality. Lost income, taxes, and the additional cost of welfare payments (only for injuries resulting in full incapacity) are the key cost drivers in both full incapacity and fatality cases.

### 3.3. Total Cost

The total cost of work-related injuries and fatalities as a result of inadequate sleep among on-call workers in Australia is estimated at $2.25 billion (Table 5). The cost of full incapacity comprises the majority of these costs (94% of total costs or $2.11 billion) with loss of earnings as the key cost driver, followed by the cost of injuries resulting in a short absence from work (6.1% of total costs or $138.32 million), and the cost of fatality (0.3% of total costs or $7.09 million).

Employers bear the largest share of the cost when injuries result in short absence from work (72% or $99.94 million). This cost mainly relates to medical compensations as part of employer excess provisions (57%), and the value of lost production and staff turnover costs such as overtime and over-employment (42%). Meanwhile, the burden of cost associated with full incapacity and fatality is largely borne by the government: 98% (2.07 billion) and 96% (6.78 million), respectively. These costs include future loss of income, welfare payments, and deadweight loss associated with tax revenue forgone.

### 3.4. Sensitivity Analysis

Table 6 provides the results of sensitivity analyses. All variations in key parameters resulted in the total cost of inadequate sleep among on-call workers ranging from $1.71 billion to $2.73 billion. The largest variance is mainly attributed to the uncertainty around the proportion of on-call workers with inadequate sleep.

## 4. Discussion

To the best of our knowledge this is the first study to quantify the economic cost of workplace injury due to inadequate sleep in on-call workers. Two-thirds of Australia’s on-call population (66%) reported obtaining inadequate sleep on weekdays (work days) and over 80% reported inadequate sleep while on-call. The resulting cost of injury is estimated at $2.25 billion per year ($1.71–2.73 billion). This equates to $1222 per person per incident involving a short-term absence from work, $2.53 million per incident classified as full incapacity, and $1.78 million for each fatality.

The annual cost of inadequate sleep due to workplace injury to on-call employees is $136.88 million ($103.70–165.91 million). Employers bear the largest share of the cost when injuries result in short absence from work (72%). This cost mainly relates to medical compensations as part of employer excess provisions (57%), and the value of lost production and staff turnover cost such as overtime and over-employment (42%). Meanwhile, the burden of cost associated with full incapacity and fatality is largely borne by the government: 98% and 96%, respectively. The annual cost of workplace injuries associated with inadequate sleep among on-call workers to the government and the economy as a whole is $2.11 billion ($1.60–2.56 billion). This includes costs in providing welfare benefits, falls in tax revenue, and costs to the health care system. Lost output alone costs the economy $1.88 billion ($1.42–2.28 billion). At a time when there is a national focus on productivity, the inescapable conclusion is that it is in the interest of both employers and the government to prioritize and invest far more in management of inadequate sleep.

The estimated cost of workplace injury reported in this study is comparable to the available national and international evidence from other populations. A recent report on inadequate sleep among the Australian general population estimated nearly 40% of Australian adults experience inadequate sleep leading to the financial loss of $26.2 billion, or $3548 per person/year [19]. A US study found that inadequate sleep among healthcare, manufacturing, and transportation workers cost $3174 per employee per year in fatigue-related productivity losses (converted to 2016 Australian dollars using AU/US PPP conversion factor of 1.50 and CPI of 1.14) [33].

The current study provided an initial insight into the potential impact of inadequate sleep of on-call workers by examining the cost of injuries classified as a short absence from work, full incapacity, or fatality. However, there are other adverse impacts of inadequate sleep for this population which have not been considered in this costing analysis. The current study did not assess the cost of inadequate sleep on well-being. In the general Australian population, well-being loss imposes an extra cost of $5419 per person annually. In addition, the impact of inadequate sleep on other household members including spouses and children has also not been considered, nor were other potential impacts from a personal or societal viewpoint. Further research is needed to understand how being called can, for example, disturb an on-call worker’s spouse’s sleep, and the subsequent cost resulting from inadequate sleep. 

Furthermore, 22% of on-call workers in the current study indicated that on-call work was not part of their primary employment (e.g., on-call role as volunteer firefighter). Therefore, the cost of injury for these individuals may be greater than estimated in the current study because an injury to these workers has the potential to impact not only their capacity in their voluntary (on-call) role but also their main place of employment, both with associated costs. Therefore, the overall cost of inadequate sleep to the individuals, their families, workplaces, government, and society in general is likely to be greater than estimated in the current study.

It is of great concern that 80% of the on-call population in this study reported obtaining inadequate sleep when on-call. Regulations in many countries, for example in Europe [34], state that on-call periods when no calls are received are just as restful as time off, despite evidence demonstrating that this is often not the case [7,35]. For time off to be fully restorative and for workers to be well-rested, it is recommended that workers be provided with the opportunity to disengage from the work [36]. To alleviate the burden of inadequate sleep, it is essential that employers recognize that on-call and time off are not equivalent when managing employees. This may involve developing work schedules which account for the demands of on-call work, particularly for instances where workers have not actually been called-in. For example, if a worker is on-call across a weekend but is not actually called, then it may be appropriate for them to have some extra time off. Furthermore, when an on-call worker obtains inadequate sleep, employers should perhaps consider additional control measures to minimize the potential fatigue-related risk.

Well-rested employees are critical to safe and productive operations, especially in on-call industries (e.g., healthcare, emergency services) where the risk of a fatigue-related error can have serious consequences to the workers themselves and the communities they serve. Our estimates of the cost of inadequate sleep in the Australian on-call workforce reinforce the importance of preventive measures to reduce fatigue-related errors. Organisations and workers are dually responsible for the management of inadequate sleep. For example, workplaces could provide sleep education to increase knowledge of how inadequate sleep can impact worker health and safety, and ways to obtain good sleep. While it is essential that workplaces provide fatigue-risk management strategies (including appropriate rosters that facilitate workers’ sleep and recovery), workers are also responsible for managing fatigue risk e.g., utilizing a sleep opportunity for sleep. Notably, across workplaces, there can be a culture of underreporting of injuries [37,38] and workers may not report sleeping poorly out of fear of a punitive response.

Determining whether sleep-related fatigue underlies or is a contributing factor to injury is often difficult, as a sleep-related fatigue injury is the endpoint of a fatigue-risk trajectory (or ‘error trajectory’) [39]. For example, the fatigued state of a worker is preceded by insufficient recovery sleep or excessive wakefulness, which could be caused by various reasons, such as failure to obtain sufficient sleep for reasons beyond the workers control (e.g., the roster did not provide an adequate sleep opportunity) or the worker choosing to engage in non-sleep activities [39]. Therefore, it is important to acknowledge that there are a multitude of factors [40] that precede a fatigue-related injury. In addition, individuals lacking sleep are often unaware of their own level of associated performance impairment [41] and therefore may not recognise lack of sleep as a contributing factor to injury. 

This study has some limitations which should be acknowledged. Firstly, self-report measures were used to estimate the prevalence of inadequate sleep among on-call workers. Self-report data allowed for a large number of on-call workers to be surveyed from various industries at low cost [42]. The literature comparing self-reported sleep duration with objective measures (actigraphy and/or polysomnography) is mixed. Some studies show that, when compared to objective measures, individuals can underestimate [43,44], or overestimate [45] total sleep duration by as much as 30–40 min. In addition, the current study exclusively utilised sleep duration as a measure of inadequate sleep. Future studies need to assess a broad range of sleep variables (such as quality and timing) to determine to what extent these factors contribute to sleep-related injury risk and subsequent economic cost. 

Given that the survey was advertised as research into the impact of on-call work on health and well-being it is possible that a disproportionate number of workers who felt that on-call work was impacting their health and well-being undertook the survey. In addition, participants were included in the study if any of their work included an on-call component, regardless of the amount of time workers were on-call. Therefore, the number and distribution (e.g., consecutive) of on-call shifts, as well as the likelihood of receiving a call (high vs. low), would likely impact upon sleep and subsequently upon the risk of injury; that is, a greater amount of on-call means a greater risk of injury. Further research is needed to determine the implications of on-call workload on injury risk. During on-call work periods, workers may not be called (i.e., not called into work). In these circumstances, there is no immediate risk of a workplace injury, because the worker is not at work. However, given that workers report inadequate sleep during on-call periods, even when no calls are received [9], if adequate sleep following an on-call shift is not obtained, the next scheduled work shift could be impacted, regardless of whether this shift is classified as ‘on-call’. Lastly, the costing analysis relied on population averages (e.g., weekly earnings, age when injury occurred). These data may mask the potential variance in economic cost per full incapacity and fatality; for example, older workers having a lower economic cost than younger workers [26]. 

## 5. Conclusions 

On-call work is often implemented in industries as it is seen to be more cost-effective for an employer to have the option of calling upon staff, rather than having employees rostered on. However, this economic benefit should be considered in the context of the high proportion of Australia’s on-call workers that are obtaining inadequate sleep, and the subsequent impact this may have on alertness, work performance, and risk of injury resulting in full incapacity or even fatality. The associated economic annual burden of over $2.53 billion is avoidable. It is the responsibility of both on-call workers and employers to implement preventive measures and reduce the prevalence of inadequate sleep in this population. 

## Figures and Tables

**Table:** 

Variable	Source	Value
A. Population of on-call workers in Australia	24% of Australia’s 9.6 million salaried workers complete on-call as part of their employment [ 4]	2,304,000
B. Proportion of on-call workers with inadequate sleep	66%; survey of on call workers (*n* = 203)	1,520,200
C. Proportion of on-call workers with work-related injuries	7.5% workplace injuries due to inadequate sleep in Australia in 2016–2017 [ 19]	114,048
D. Resulting in short absence from work	[C-E-F]	113,211
E. Resulting in full incapacity	Based on Deloitte Access Economics (2011) (e.g., in this study it was estimated that 0.73% of all motor vehicle accidents resulted in injury sufficient that the individual does not return to work) [ 19]. In this study, this proportion (0.74%) was used as a proxy of the number of cases resulting in full incapacity due to inadequate sleep.	833
F. Resulting in fatality	0.04 mortality rate per 1000 attributed to workplace injuries condition [ 19]	4

**Table 1:** Economic cost borne by the employer, worker, and government.

Conceptual Group	Cost Item	Employer	Worker	Government
Production disturbance costs	Value of lost production	Overtime premium and value of wages paid while away from work	Zero	Zero
	Staff turnover costs	Staff turnover costs	Zero	Zero
Human capital costs	Net present value of lost earnings	Zero	Zero	Loss of income and welfare payments transferred to worker for loss of wage minus deadweight loss associated with tax revenue forgone
Medical costs	Medical and rehabilitation costs	Threshold medical payments	Gap payments	Medical payments not covered by employer or worker
Admin. costs	Investigation costs	Employer investigation costs	Zero	Costs of running the compensation system (including investigation claims)
	Travel costs	Zero	Out of pocket expenses	Compensation for travel costs
	Funeral costs	Zero	Out of pocket expenses	Zero
Other	Carers	Zero	Zero	Payments to carers
	Aids, equipment, and modifications	Zero	Zero	Reimbursements for aids, equipment, and modifications
	Postvention	Postvention	Zero	Postvention
Transfer costs	Deadweight costs of tax revenue foregone	Zero	Zero	Deadweight costs of tax revenue foregone

Source: Safe Work Australia, 2015 [24].

**Table 2:** Summary of key assumptions.

Incidents Indicator Data	Source	Value
Mean age of incident	Average age of on-call workers obtained from Survey Monkey	42
Average life expectancy at birth	Australian Bureau of Statistics (ABS) Deaths, Australia, 2014 [ 29]	82.4
Potential years of life lost (YLL)	(Average life expectancy at birth [ 29]−Mean age of incident)	40.4
Potential productive years of life lost (PYLL)	(Average retirement age in Australia−Mean age of incident)	24.0
**Costing Analysis**		
Average earnings	Proxy for productivity, ABS 6306.0 Employee Earnings and Hours survey, May 2016: Average weekly earnings as a weighted earnings for full time (69%) and part time (31%) employees	$1317
Discount rate	Opportunity cost of money: Average of rates of return for private and government saving instruments and Reserve Bank of Australia (RBA) target for March 2005 to May 2016 [ 30]	4.41%
Inflation rate	Average of annual weighted ABS 6401.0 Consumer Price Index (CPI) from December 2004 to June 2016 [ 28]	2.58%
Productivity rate	Annual increase in workers’ productivity. Safe Work Australia report 2012–2013 [ 24]	1.75%
Average tax for foregone earnings	Australian Taxation Office [ 31]	25.00%
Transfer costs	Deadweight cost of welfare payments and tax losses. Safe Work Australia report 2012–2013 [ 24]	28.75%
Welfare for disability per year	Disability support pension $877.10 per fortnight [ 32]	$22,805
Carers—full incapacity cases only from incident to end of life	Estimated carer allowance of $118.20 per fortnight paid to someone who provides daily care and attention to someone in their own home adjusted to 2016 dollars using CPI correction factor [ 28]. Department of Social Services, 2013	$2837
Aids and modifications—full incapacity cases only from incident to end of life	Estimated applicable Disability Support Pension payments of $680 per annum, discounted to present value over the period between the incident and reduced life expectancy adjusted to 2016 dollars using CPI correction [ 28]. Safe Work Australia report 2012–2013 [24]	$680

**Table 3:** Self-reported sleep duration by Australian on-call workers.

**a. How Much Sleep Do You Regularly Get on Weekdays (or Work Days), Including Naps?**		
**Sleep Duration**	**Number of Respondents**	**Percentage (%)**
<5 h	13	6
5 h–6 h	50	24
6 h–7 h	76	36
7 h–8 h	55	26
8 h–9 h	13	6
>9 h	4	2
Total	211	100
**b. How Many Hours of Sleep Do You Regularly Get When On-Call, Including Naps?**		
**Sleep Duration**	**Number of Respondents**	**Percentage (%)**
<5 h	32	15
5 h–6 h	68	32
6 h–7 h	68	32
7 h–8 h	37	18
8 h–9 h	6	3
>9 h	0	0
Total	211	100

**Table 4:** Average cost of work-related injuries resulting in short absence, full incapacity, and fatality among on-call workers (in 2016 Australian dollars).

Cost Category	Short Absence	Full Incapacity	Fatality
Production disturbance costs	$369	$42,327	$42,327
Human capital costs	$0	$2,248,987	$1,573,132
Medical costs	$820	$12,515	$2430
Administrative costs	$33	$2634	$7030
Other	$0	$104,213	$30,000
Transfer costs	$0	$121,825	$121,825
Average cost per person	$1222	$2,532,502	$1,776,744

**Table 5:** Total cost of work-related injuries resulting in short absence, full incapacity, and fatality among on-call workers (in 2016 Australian dollars).

Cost Category	Short Absence	Full Incapacity	Fatality	Overall
Production disturbance costs	$41,746,970	$35,239,760	$168,958	$77,155,688
Human capital costs	$0	$1,872,395,143	$6,279,439	$1,878,674,582
Medical costs	$92,833,395	$10,419,368	$9700	$103,262,464
Administrative costs	$3,735,978	$2,192,938	$28,062	$5,956,977
Other	$0	$86,762,664	$119,750	$86,882,414
Transfer costs	$0	$101,425,480	$486,287	$101,911,766
Total	$138,316,343	$2,108,435,353	$7,092,195	$2,253,843,892
Employer	$99,937,659	$36,644,273	$296,372	$136,878,304
Worker	$5,717,179	$1,608,696	$17,502	$7,343,376
Government	$32,661,506	$2,070,182,385	$6,778,322	$2,109,622,212

**Table 6:** Sensitivity analysis of key parameters (in 2016 Australian dollars).

Parameter Varied	Cost of Inadequate Sleep
Proportion of on-call workers with inadequate sleep	
Sensitivity 1 = 50%	$1,707,457,494
Baseline = 66%	$2,253,843,892
Sensitivity 2 = 80%	$2,731,931,990
Proportion of injuries resulting in short absence	
Sensitivity 3 = 6.5%	$1,953,331,373
Baseline = 7.5%	$2,253,843,892
Sensitivity 4 = 8.5%	$2,554,356,411
Proportion of injuries resulting in full incapacity	
Sensitivity 5 = 0.6%	$1,878,550,243
Baseline = 0.7%	$2,253,843,892
Sensitivity 6 = 0.8%	$2,455,925,087
Rate of injuries resulting in fatality per 1000	
Sensitivity 7 = 0.02	$2,250,806,470
Baseline = 0.04	$2,253,843,892
Average age of injury	
Sensitivity 8 = 40	$2,376,206,296
Baseline = 42	$2,253,843,892
Average life expectancy at birth	
Sensitivity 9 = 71.6	$2,128,392,304
Sensitivity 10 = 77.0	$2,188,533,859
Baseline = 82.4	$2,253,843,892
Discount rate used to convert future costs to present value	
Sensitivity 11 = 3%	$2,716,424,664
Baseline = 4.4%	$2,253,843,892
Sensitivity 12 = 5%	$2,099,113,411
Average earnings	
Sensitivity 13 = $1231	$2,156,246,394
Baseline = $1317	$2,253,843,892
